# Xenon: A Solution for Anesthesia in Liver Disease?

**DOI:** 10.5812/hepatmon.8437

**Published:** 2012-11-20

**Authors:** Ali Dabbagh, Samira Rajaei

**Affiliations:** 1 Anesthesiology Research Center, Shahid Beheshti University of Medical Sciences, Tehran, IR Iran; 2 School of Allied Medicine, Tehran University of Medical Sciences, Tehran, IR Iran

**Keywords:** Xenon, Liver, Anesthesia

There are reports that demonstrate the Avicenna to be the first anesthesiologist using modern methods of anesthesia and describing them in his Canon textbook (1); however, the majority of clinicians of worldwide believe that the modern practice of anesthesia is not too much old since 1846 and has affected the fate of surgical era after its invention in 1846 (2, 3). From the first days, anesthesia has been based on a limited number of pharmaceuticals and among them, anesthetic gases have a major role (4). The anesthetic gases have reached a great development; reaching the latest versions of halogenated hydrocarbons; sevoflurane being the last and possibly one of the best members of this family after decades (3-5). However, each pharmaceutical might have some potential side effects and the volatile anesthetics are not exception for this fact; one of their main drawbacks is the possibility of volatile agent that induced hepatic injury (2-6). Though its incidence has declined dramatically after introduction of newer halogenated hydrocarbons like sevoflurane and desflurane, it has not vanished totally and studies regarding different effects of these gases are among the current research priorities (7-9). So, there is a widespread search for finding safer anesthetic gases with fewer side effects. Xenon, a member of the noble gas family, was discovered for the first time in 1898 (10). However, its anesthetic properties were first described at 1939 (10) and it received more intense attention at 1951 when anesthetic properties of xenon and krypton were reported in Science Journal (11, 12). But its clinical use was not so much a matter of interest; then, it took about 40 years to rediscover it for any possible clinical application (13, 14). The mechanism of xenon anesthesia is its effect on the N-methyl-D-aspartate receptors through noncompetitive inhibition (10). At the present time, there is a growing enthusiasm between clinicians to use it as an anesthetic; since it has “nearly all” the characteristics of an ideal anesthetic gas (15-20). However, it has two main drawbacks: very high price (13, 21, 22) and the problems related to drug scavenging (2, 10, 23). Technology has improved and the more newer technologies used in updated anesthesia machines has helped us to decrease the waste of gas; so gas wastage has decreased both needed dose and the scavenging needs (2, 10, 13, 21). In most circumstances, xenon is an inert gas, producing effective inhalational anesthesia under norm baric condition (24-30); having “nearly all” the necessary features of an ideal anesthetic gas (16-20) producing a very steady state regarding the left ventricular function, with ulmost cardiovascular stability and fewer “fluctuations of cardiovascular system parameters”; only some right ventricular function impairment has been noted (14, 18, 31, 32). Also, xenon does not have “negative inotropic effects and vasodilatation” which is a very important and useful characteristic for patients with depressed cardiovascular function and are not hemodynamically stable; with very low incidence of toxicity or teratogenicity (22). At the same time, in clinical conditions, has been shown to exert protection of brain or myocardium from ischemia-reperfusion injury (14, 33, 34). These protective effects are of course, among all the members of the inert gas family, it is the only clinical agent having anesthetic actions (14). But the organ protective effect of noble gas family members is not limited to xenon; it should be said that xenon is the only member of the family having organ protection effects and clinical anesthetic effects (14, 24). For example, helium has organ protecting effects without anesthetic effects (24). There are many studies regarding the effects of xenon as an anesthetic on the liver; directly or indirectly; some being animal and others clinical studies (2, 10, 21, 22, 31, 34-39). Xenon anesthesia helps us prevent liver exposure to halogenated anesthetic gases; so the liver cells are not exposed to these agents; while xenon has not demonstrated any possible liver cell injury but it possibly would protects these cells (2); especially when considering the organ-protective effects of xenon (37). Also, xenon anesthesia has much better results regarding the tumor size after laparoscopy in rats compared with CO2, decreasing the size of tumor after using helium or xenon pneumo-peritoneum possibly by their inflammatory modification effects (35). Xenon anesthesia has been shown to produces “the highest regional blood flow in the brain, liver, kidney and intestine” (22); which is a very interesting effect for patients undergoing liver surgeries like liver transplant (22, 23) which could induce a “feasible” anesthesia for liver transplant with satisfactory situation in the “immediate postoperative liver function” (34). Also, xenon causes the flow of the hepatic artery to remain stable with no vivid “change in the hepatic arterial buffer responses” (31). Also, it was demonstrated that xenon anesthesia could reduce “plasma catecholamine concentrations” which would result in improved nitrogen balance after surgery (38). The patients undergoing anesthesia with xenon for liver surgeries tolerate less fluctuations in “liver circulation parameters” so, the perfusion status of the liver is much better preserved with xenon anesthesia. We still need more studies to see whether we could decrease the costs of xenon anesthesia by developing newer anesthesia machines decreasing wastage of anesthetics to the environment; in such a way that its many benefits could overcome the costs. The primary studies have demonstrated that improved circulatory equipments could improve the clinical outcome when using xenon anesthesia (17). Helium when used instead of carbon dioxide for laparoscopy could decrease the size of tumor after surgery (35). Except for xenon, krypton and argon are also potential anesthetic gases (13). Even, krypton has been shown to have better results in animal studies; while not yet proved in clinical studies. Finally, at the present time, xenon is used limitedly in European and the US for some very critical patients like hepatic transplantation; however, using its beneficial effects instead of the routine volatile agents depends on the improvement of new recycling anesthesia machines with rebreathing system properties providing efficient cost containment approaches (13, 35).
